# Type M Resistance to Macrolides Is Due to a Two-Gene Efflux Transport System of the ATP-Binding Cassette (ABC) Superfamily

**DOI:** 10.3389/fmicb.2018.01670

**Published:** 2018-07-31

**Authors:** Francesco Iannelli, Francesco Santoro, Maria Santagati, Jean-Denis Docquier, Elisa Lazzeri, Gabiria Pastore, Marco Cassone, Marco R. Oggioni, Gian M. Rossolini, Stefania Stefani, Gianni Pozzi

**Affiliations:** ^1^Department of Medical Biotechnologies, University of Siena, Siena, Italy; ^2^Section of Microbiology, Department of Biomedical and Biotechnological Sciences, University of Catania, Catania, Italy

**Keywords:** ABC transporter, macrolide efflux, Φ1207.3, prophage, *mef*(A), *msr*(D), *Streptococcus pneumoniae*, *Streptococcus pyogenes*

## Abstract

The *mef*(A) gene was originally identified as the resistance determinant responsible for type M resistance to macrolides, a phenotype frequently found in clinical isolates of *Streptococcus pneumoniae* and *Streptococcus pyogenes*. MefA was defined as a secondary transporter of the major facilitator superfamily driven by proton-motive force. However, when characterizing the *mef*(A)-carrying elements Tn*1207.1* and Φ1207.3, another macrolide resistance gene, *msr*(D), was found adjacent to *mef*(A). To define the respective contribution of *mef*(A) and *msr*(D) to macrolide resistance, three isogenic deletion mutants were constructed by transformation of a *S. pneumoniae* strain carrying Φ1207.3: (i) Δ*mef*(A)–Δ*msr*(D); (ii) Δ*mef*(A)–*msr*(D); and (iii) *mef*(A)–Δ*msr*(D). Susceptibility testing of mutants clearly showed that *msr*(D) is required for macrolide resistance, while deletion of *mef*(A) produced only a twofold reduction in the minimal inhibitory concentration (MIC) for erythromycin. The contribution of *msr*(D) to macrolide resistance was also studied in *S. pyogenes*, which is the original host of Φ1207.3. Two isogenic strains of *S. pyogenes* were constructed: (i) FR156, carrying Φ1207.3, and (ii) FR155, carrying Φ1207.3/Δ*msr*(D). FR155 was susceptible to erythromycin, whereas FR156 was resistant, with an MIC value of 8 μg/ml. Complementation experiments showed that reintroduction of the *msr*(D) gene could restore macrolide resistance in Δ*msr*(D) mutants. Radiolabeled erythromycin was retained by strains lacking *msr*(D), while *msr*(D)-carrying strains showed erythromycin efflux. Deletion of *mef*(A) did not affect erythromycin efflux. This data suggest that type M resistance to macrolides in streptococci is due to an efflux transport system of the ATP-binding cassette (ABC) superfamily, in which *mef*(A) encodes the transmembrane channel, and *msr*(D) the two ATP-binding domains.

## Introduction

Macrolides are antibiotic compounds composed of 14 (erythromycin and clarithromycin), 15 (azithromycin), or 16 (josamycin, spiramycin, tylosin)-membered lactones to which amino and/or neutral sugars are linked (Roberts et al., 1999; Roberts, 2008). Resistance to macrolides in streptococci is usually associated with two major mechanisms: (i) target site modification, arising from the presence of *erm*(B) or *erm*(A) subclass *erm*(TR) belonging to the class of *erm* (erythromycin ribosome methylation) methylase genes (Leclercq and Courvalin, 1991; Seppala et al., 1998) and (ii) drug efflux, associated to the presence of *mef*(A) (macrolide efflux) genes (Clancy et al., 1996; Sutcliffe et al., 1996; Tait-Kamradt et al., 1997). Methylation of 23S rRNA causes a reduced binding to macrolide, lincosamide, and streptogramin B antibiotics (MLS_B_ phenotype), whereas active efflux of macrolides confers a low level of resistance to resistance only to 14- and 15-membered macrolides, but not to 16-membered macrolides, lincosamides, and streptogramin B antibiotics (M phenotype; Weisblum, 1995; Roberts et al., 1999).

The macrolide efflux *mef*(A) gene was originally described in *Streptococcus pyogenes* (Clancy et al., 1996) while the allelic variant *mef*(E) was first described in *Streptococcus pneumoniae* (Tait-Kamradt et al., 1997). These variants are highly homologous (about 90% nucleotide identity) and are grouped in the same *mef*(A) class of macrolide resistance genes (Roberts et al., 1999). The *mef*(A) gene was not found only in *S. pyogenes* and *S. pneumoniae*, but also in a wide variety of other streptococcal species such as *Streptococcus agalactiae, Streptococcus mitis, Streptococcus oralis*, and *Streptococcus salivarius*, in other Gram-positive genera including *Corynebacterium, Enterococcus, Micrococcus*, and *Staphylococcus*, and in Gram-negative genera such as *Acinetobacter, Bacteroides*, and *Neisseria* (Roberts et al., 1999; Klaassen and Mouton, 2005; Santagati et al., 2009). In some countries, the *mef*(A) gene has become more common than *erm*(B) in macrolide-resistant *S. pneumoniae* and *S. pyogenes* (Green et al., 2006; Reinert et al., 2006; Rudolph et al., 2013) and it has been used as a marker for molecular epidemiology studies (Daly et al., 2004; Klaassen and Mouton, 2005; Amezaga and McKenzie, 2006). The *mef*(A) allelic variants are carried by different genetic elements. In *S. pneumoniae*, we described a 7244-bp non-conjugative element named Tn*1207.1* carrying *mef*(A), whereas *mef*(E) was found to be carried by the 5532-bp pneumococcal genetic element (mega; Santagati et al., 2000; Gay and Stephens, 2001; Del Grosso et al., 2002, 2006). In *S. pyogenes, mef*(A) is carried by Φ1207.3, a 52,491-bp prophage which we found in the erythromycin-resistant clinical strain 2812A, transferable to a variety of streptococcal species and whose left 7244-bp sequence is 100% identical to pneumococcal Tn*1207.1* (Santagati et al., 2003; Pozzi et al., 2004; Iannelli et al., 2014a). Other *mef*(A)-carrying prophages in clinical isolates of *S. pyogenes* include Φ10394.4 (Banks et al., 2003), Φm46.1, and its variant VP_00501.1 (Brenciani et al., 2004, 2010; Vitali et al., 2016).

At first, Mef(A) was defined as a secondary transporter of the major facilitator superfamily (MFS)^1^ and considered to be the only gene product responsible for type M macrolide resistance, as shown by cloning and functional characterization in *Escherichia coli* (Clancy et al., 1996). However, in *mef*(A)-carrying genetic elements such as Tn*1207.1* and Φ1207.3, another macrolide resistance gene, *msr*(D), was always found adjacent to *mef*(A), and *msr*(D) was shown to contribute to the macrolide efflux resistance of streptococcal strains carrying the *mef*(A)*–msr*(D) tandem pair (Iannelli et al., 2004; Ambrose et al., 2005; Vitali et al., 2016; Zhang et al., 2016; Tatsuno et al., 2018).

To assess the relative contribution of *mef*(A) and *msr*(D) to macrolide efflux, we generated in-frame isogenic mutants of *S. pneumoniae* and *S. pyogenes* carrying *mef*(A)-*msr*(D) to be used in functional studies. Mutant strains were tested by (i) determining the minimal inhibitory concentration (MIC) of erythromycin and (ii) assaying the actual intracellular accumulation of radiolabeled erythromycin. Results of functional studies were in accordance with bioinformatics analysis predicting that the tandem *mef*(A)*-msr*(D) gene pair encodes an efflux transport system of the ATP-binding cassette (ABC) superfamily.

## Materials and Methods

### Bioinformatic Softwares

Protein sequence analysis was performed with the softwares Phyre2 (Kelley et al., 2015) and TMpred. Conserved aminoacids were identified with the PSI-BLAST multiple sequence alignment. Terminator sequence was predicted with RNAstructure ver. 5.02 (Mathews Lab). Nucleotide sequence analysis was performed using Microbial Nucleotide BLAST with the Megablast algorithm^2^.

### Bacterial Strains and Growth Conditions

Streptococcal strains used in this work and their relevant properties are reported in **Table 1**. Bacteria were routinely grown in Tryptic Soy Broth (TSB) or Tryptic Soy Agar (Difco) supplemented with 3% horse blood at 37°C. When required, 500 μg/ml streptomycin, 10 μg/ml novobiocin, 0.5 μg/ml erythromycin, 100 μg/ml spectinomycin, and 3 μg/ml chloramphenicol were added to both liquid and solid media.

**Table 1:** Bacterial strains.

Strain	Relevant properties^a^	Origin (references)
*S. pneumoniae*		
D39	Type 2 Avery’s strain	Pearce et al., 2002; Lanie et al., 2007
Rx1	Unencapsulated derivative of D39	Smith and Guild, 1979; Pearce et al., 2002
DP1004	Unencapsulated derivative of D39; *str-41*; Sm^R^	Salles et al., 1992; Iannelli and Pozzi, 2004
FP11	Rx1 unencapsulated competence deficient derivative, Δ*comC*; Nov^R^, Cm^R^	Iannelli and Pozzi, 2007; Santoro et al., 2010
FR122	FP11 derivative carrying Φ1207.3; Nov^R^, Cm^R^, Em^R^	This study
FR125	Rx1 unencapsulated competence deficient derivative carrying Φ1207.3; Δ*comC, str-41*, Δ*celB*; Cm^R^, Sm^R^, Spc^R^, Em^R^	Iannelli and Pozzi, unpublished results
FR183	DP1004 derivative carrying Φ1207.3 (by transformation with FR125 chromosomal DNA); Sm^R^, Em^R^	This study
FP39	FR183 derivative, Δ*mef*(A) and Δ*msr*(D); Sm^R^, Cm^R^	This study
FP40	FR183 derivative, Δ*mef*(A); Sm^R^, Em^R^, Cm^R^	This study
FP59	FR183 derivative, Δ*msr*(D); Sm^R^, Spc^R^	This study
FP92	FP59 derivative, *msr*(D) (by transformation with a *msr*(D)-containing PCR fragment amplified from FR183); Sm^R^, Em^R^	This study
FR139	FP11 derivative carrying Φ1207.3 – Δ*msr*(D) (by conjugation with FP59); Nov^R^, Spc^R^	This study
*S. pyogenes*		
D471	Streptomycin-resistant serotype M6 strain from RU collection, Sm^R^	Scott and Fischetti, 1983
FR156	D471 derivative carrying Φ1207.3 (by conjugation with FR122); Sm^R^, Em^R^	This study
FR155	D471 derivative carrying Φ1207.3 – Δ*msr*(D) (by conjugation with FR139); Sm^R^, Spc^R^	This study
FR160	FR155 derivative carrying Φ1207.3 (by conjugation with FR122); Sm^R^, Em^R^, Spc^R^	This study

*^*a*^*nov-1* and *str-41* indicate point mutations conferring resistance to novobiocin and streptomycin, respectively. Nov, novobiocin; Sm, streptomycin; Cm, chloramphenicol; Tc, tetracycline; Em, erythromycin; Spc, spectinomycin; Km, kanamycin.*

### Construction of the Isogenic *S. pneumoniae* Mutant Strains

To allow construction of mutants deleted for the macrolide resistance genes object of this study, and to facilitate further analysis, it was essential to work in a *S. pneumoniae* strain which is readily transformable and whose genomic sequence is available (Hoskins et al., 2001). For this reason, we transferred Φ1207.3 to the chromosome of unencapsulated laboratory strains of *S. pneumoniae* which are derived from type 2 strain D39 (Iannelli et al., 1999; Pearce et al., 2002).

Integration of Tn*1207.1* or Φ1207.3 into the chromosome of *S. pneumoniae* occurs within the cds of *celB*, a gene involved in DNA uptake during transformation, and disruption of *celB* leads to impairment of competence for genetic transformation in *S. pneumoniae* strains carrying Tn*1207.1* or Φ1207.3 (Santagati et al., 2000, 2003). For this reason, strain FR125 was constructed in which *celB* was deleted and integration of Φ1207.3 after mating occurred at a different chromosomal location (Iannelli and Pozzi, unpublished results). Subsequently, strain FR183, a transformable *S. pneumoniae* DP1004 derivative strain carrying Φ1207.3 integrated elsewhere than *celB*, was obtained by transformation with the chromosomal DNA purified from FR125 (**Table 1**).

### Matings

Mating experiments were performed as already described (Iannelli and Pozzi, 2007). Briefly, cells were grown separately in presence of appropriate antibiotics until the end of exponential phase (OD_590nm_ = 0.8). Cells were mixed at 1:10 ratio (Donor/Recipient), harvested by centrifugation and plated. Following 4-h incubation, cells were harvested by scraping the plates. Selection of recombinants was carried out with a multilayer plating procedure. Transfer frequencies were determined by plating alone each parent strain that was also checked for spontaneous antibiotic resistance acquisition.

### Oligonucleotide Primers

Oligonucleotide primers used for mutagenesis, sequencing, and PCR selection of recombinant strains are listed in Supplementary Table S1.

### PCR Mutagenesis

Gene splicing by overlap extension (gene SOEing) was used to generate the mutagenic constructs for gene deletions as already described (Pearce et al., 2002; Iannelli and Pozzi, 2004). The deletion of *mef*(A) and *msr*(D) genes was obtained with a mutagenic construct containing the *ami*/*cat* cassette flanked at the left by a 195-bp DNA fragment corresponding to nucleotides 3112–3307 of Φ1207.3 and at the right by a fragment corresponding to the 532 nucleotides located downstream of the *msr*(D) stop codon. The *ami*/*cat* chloramphenicol-resistance cassette was obtained with the primer pair IF38/IF39 using the *E. coli* pEVP3 plasmid as template (Claverys et al., 1995). The flanking regions were amplified, respectively, with the primer pairs IF105/F35 and IF183/IF175 from chromosomal DNA of FR183. To minimize possible polar effects, the *mef*(A) coding sequence (cds) was deleted in-frame by allelic replacement with the cds of the *cat* chloramphenicol-resistance gene. The *cat* cds was amplified with primer pair IF184/IF39 from a Ω*cat*(pC194)-carrying strain (Iannelli et al., 2014b) and flanked by a 1116-bp DNA region located upstream *mef*(A) start codon and a DNA fragment spanning the 449 nucleotides downstream of the *mef*(A) stop codon, obtained, respectively, with the primer pairs IF176/IF182 and IF185/F20 from FR183 chromosome. A genetic construct containing the *ami*/*spc* cassette flanked by the 281-bp and 532-bp DNA fragments located respectively upstream and downstream of *msr*(D) cds was produced to inactivate *msr*(D). The primer couple IF100/IF101 was used to amplify the spectinomycin-resistance cassette from the pR412 plasmid (Martin et al., 2000). This cassette was flanked by the *msr*(D) flanking segments amplified with the primer pair IF171/IF174 from FR183 chromosome. Linear PCR constructs were used directly as donor DNA in transformation experiments. Mutant strains were selected for acquisition of chloramphenicol or spectinomycin resistance and the desired mutations were confirmed by sequencing. PCR-based selection of *S. pneumoniae* complemented strains was carried out with the primers pair IF169/IF170 according to the protocol already described (Iannelli and Pozzi, 2004).

### Erythromycin Sensitivity Determination

The MIC was assessed by microdilution techniques as suggested by the (Clinical and Laboratory Standards Institute [CLSI], 2017). Briefly, MICs were determined as follows: bacterial cells were grown until exponential growth phase (OD_590nm_ = 0.25) in TSB medium and stored at -70°C in 10% glycerol, then frozen cultures were thawed and diluted at 5 × 10^4^ CFU/ml in TSB broth containing serial twofold dilutions of antibiotic, and incubated at 37°C for 18 h. Bacterial growth was determined turbimetrically using the microplate ELISA reader VERSAmax (Molecular Devices). Results are presented as the geometric mean and are derived from at least three experiments. *S. pneumoniae* ATCC49619 was used as reference control strain as recommended (Clinical and Laboratory Standards Institute [CLSI], 2017).

### Erythromycin Efflux Assay

Frozen starter culture was diluted 100-fold in 100 ml warm TSB medium and grown at 37°C until an optical density at 590 nm of 0.25. Radiolabeled erythromycin [N-methyl-^14^C] (50 μCi/mmol, purchased from Bio Trend, Germany) was added at a final concentration of 0.2 μg/ml (Sutcliffe et al., 1996). Erythromycin uptake was assessed by taking 5-ml samples from each culture every 10 min for 40 min after the addition of [^14^C]erythromycin, filtering samples with prewet GF/C glass microfiber filters (Whatman), and washing the filters twice with 5 ml of a 0.9% NaCl and 1 μg/ml erythromycin solution (Sutcliffe et al., 1996). Filters were dried O.N., dissolved in 10 ml of Insta-Gel Plus liquid (Packard) and the [^14^C] erythromycin cell-associated was counted with a TRI-CARB 2000 CA Liquid Scintillation Analyzer (Packard).

## Results

### *mef*(A) and *msr*(D) Encode an ABC Transport System

**FIGURE 1:**
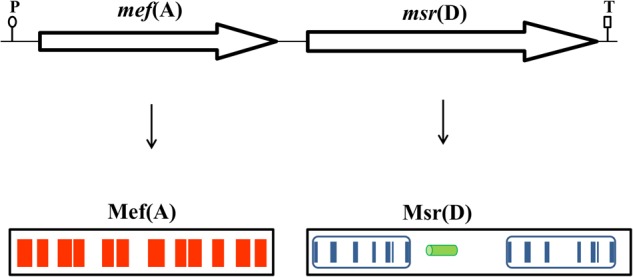
The *mef*(A)–*msr*(D) operon is 3290-bp-long and includes a single promoter sequence (P) and a terminator sequence (T). Mef(A) contains 12 transmembrane domains spanning the whole protein (red bars). Msr(D) contains two nucleotide binding domains (NBDs, blue boxes) connected by an alpha helical structure (green cylinder). Each NBD has seven motifs (blue bars): (1) the “A-loop” that provides an aromatic side chain residue that interacts with the adenine ring of bound nucleotide (phenylalanine in both NBDs); (2) the “Q-loop” involved in the interaction with the transmembrane domain and with the γ-phosphate through a water bond; (3) the “P-loop” (or “Walker-A” motif) that binds the nucleotide; (4) the “LSGGQ motif” (also called “C-loop” or “ABC signature motif”) which contacts the nucleotide in the ATP-bound state; (5) the “Walker-B” motif that has a conserved glutamate residue that orchestrates the nucleophilic attack on ATP via a water molecule; (6) the “D-loop” involved in the contact between the two NBDs through an interaction with the Walker-A asparagine residue; and (7) the “switch motif” that contains a histidine side chain thought to contribute to the catalytic reaction. Both C-loop motifs of Msr(D), MSGGE and LSGGE, are not identical to the consensus (LSGGQ).

In clinical isolates of macrolide resistant streptococci, the macrolide efflux gene, *mef*(A), is found in tandem with *msr*(D), a gene which encodes for an ATP-binding transporter (Santagati et al., 2000, 2003; Gay and Stephens, 2001; Banks et al., 2003; Brenciani et al., 2004). Bioinformatic analysis of the prototypic tandem *mef*(A)-*msr*(D) from Tn*1207.1* (Santagati et al., 2000) and their flanking regions revealed that they constitute a two-gene operon which is 3290 bp long, with only one putative promoter sequence -35 TTGCTT, extended -10 TGTGTTAAAAT (nucleotides 2890–2895 and 2908–2918, respectively, Genbank AF227520) located upstream of the *mef*(A) start codon and a single predicted terminator sequence (nucleotides 6143–6179, Genbank AF227520) located downstream of the *msr*(D) stop codon. As previously demonstrated (Ambrose et al., 2005; Chancey et al., 2015), RT-qPCR analysis confirmed that *mef*(A) and *msr*(D) genes were part of a single transcriptional unit (data not shown). Sequence analysis of the Mef(A) protein with TMpred showed the presence of 12 predicted transmembrane domains, which have the potential to form six transmembrane helices (**Figure 1**). When the protein sequence of Msr(D) was analyzed by the PSI-BLAST multiple sequence alignment, it was possible to show the presence of two nucleotide binding domains (NBDs) typical of ATP-binding transporters (spanning amino acids 12–154, and 304–463, Genbank AAG12999.1), and a long predicted alpha helical structure (spanning amino acids 181–227) between the two NBDs (**Figure 1**). Altogether, *mef*(A) and *msr*(D) appear to constitute a two-gene efflux transport system of the ABC superfamily, *mef*(A) encodes the pore (transmembrane channel), and *msr*(D) the two ATP-binding domains.

### *mef*(A) and *msr*(D) Are Found in Tandem in Bacterial Genomes

The *mef*(A) gene sequence (GenBank AF227520) was used as a query to interrogate the database of 20,187 complete Microbial genomes (accessed in May 2018). The *mef*(A) gene was found in 37 genomes, of those: 29 were streptococcal genomes and eight belonged to other genera (namely *Turicibacter, Clostridium, Enterococcus, Clostridioides, Gardnerella*, and *Bacillus*). In 33 out of 37 cases, the *mef*(A) gene was in tandem with *msr*(D), while in the remaining four cases, *msr*(D) was substituted by a gene coding for a putative nucleotidyltransferase. No information on macrolide resistance was available for these four genome strains.

### Type M Macrolide Resistance Depends on the ATP-Binding Transporter Msr(D)

**FIGURE 2:**
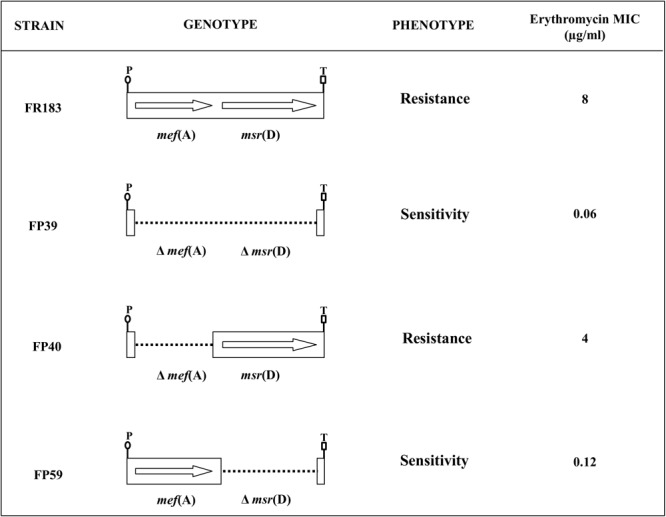
The *mef*(A)-*msr*(D) operon, carried by Φ1207.3, confers type M resistance to macrolides. Isogenic mutants were produced in a Φ1207.3-carrying *S. pneumoniae* strain. CDSs were deleted by allelic replacement with mutagenic antibiotic resistance cassettes. In order to minimize polar effect, *mef*(A) CDS was in frame deleted with the chloramphenicol resistance *cat* CDS. The genotype and relative erythromycin resistance phenotype of the pneumococcal isogenic strains are schematized. The genes are reported as arrows while dotted lines indicate gene deletions; the transcriptional promoter (P) and the putative transcriptional terminator (T) of the operon are reported.

To define the respective contribution of *mef*(A) and *msr*(D) gene products to macrolide efflux resistance, isogenic deletion mutants were constructed in *S. pneumoniae* for the *mef*(A)–*msr*(D) tandem pair carried by two different genetic elements (**Figure 2**). Three isogenic deletion mutants were constructed in the Φ1207.3-carrying strain FR183: (i) FP39, Δ*mef*(A)–Δ*msr*(D); (ii) FP40, Δ*mef*(A); and (iii) FP59, Δ*msr*(D). In FP39, a 2748-bp DNA fragment (position 3308–6055, GenBank AY657002) containing *mef*(A) and *msr*(D) was deleted and replaced with the 850-bp *ami/cat* cassette. In FP40, a 1218-bp DNA fragment (position 3255–4472, GenBank AY657002) containing the cds of *mef*(A) was deleted and replaced in-frame with the 651-bp cds of the *cat* gene. In FP59, a 1464-bp DNA fragment (position 4592–6055, GenBank AY657002) containing the *msr*(D) cds was deleted and replaced with the 894-bp *ami/spc* cassette (**Figure 2** and **Table 1**).

Sensitivity to erythromycin of the *S. pneumoniae* strains carrying *mef*(A)–*msr*(D) and of their isogenic mutants was tested in liquid medium. It was clearly shown that *msr*(D) is required for macrolide resistance (**Figure 1** and **Table 2**). Deletion of *mef*(A) produced only a twofold reduction of the MIC, while deletion of *msr*(D) produced a 64-fold MIC decrease (8 to 0.12 μg/ml), and when both genes were deleted, the MIC decreased 128-folds (**Table 2**). Identical results were also obtained using the allelic variants of *mef*(A) and *msr*(D) originally found in a macrolide-resistant clinical strain of *S. pneumoniae* formerly described as a “*mef*(E)-positive isolate” (GenBank AF376746) (Del Grosso et al., 2002, 2004). Deletion mutants were constructed and tested for erythromycin resistance. Results on MIC reduction were identical to those observed for the *mef*(A)–*msr*(D) alleles described above (data not shown).

**Table 2:** Sensitivity to erythromycin.

Strain	Genotype	MIC of erytromycin (μg/ml)^a^	Phenotype
*Streptococcus pneumoniae*			
FR183	*mef*(A), *msr*(D)	8	Resistant
FP39	Δ*mef*(A), Δ*msr*(D)	0.06	Sensitive
FP40	Δ*mef*(A), *msr*(D)	4	Resistant
FP59	*mef*(A), Δ*msr*(D)	0.12	Sensitive
DP1004^b^	–	0.03	Sensitive
*Streptococcus pyogenes*			
FR156	*mef*(A), *msr*(D)	8	Resistant
FR155	*mef*(A), Δ*msr*(D)	0.12	Sensitive
D471^b^	–	0.03	Sensitive

*^*a*^MIC interpretative standard: sensitive ≤ 0.25 μg/ml, intermediate = 0.5 μg/ml, and resistant ≥ 1 μg/ml.*

*^*b*^DP1004 and D471 are parental erythromycin sensitive strains used as controls.*

### Complementation of the *msr*(D) Deletion

To confirm the role of *msr*(D) in determining type M resistance to macrolides, deletion of *msr*(D) was complemented in *S. pneumoniae* FP59 carrying Φ1207.3/Δ*msr*(D). Competent cells of FP59 were transformed with a 2206-bp PCR fragment containing *msr*(D) obtained from wild type Φ1207.3, using primer pair IF171/IF175. Transformants were selected by PCR for the presence of the *msr*(D) gene, as previously described (Iannelli and Pozzi, 2004). A total of four transformants were selected out of 186 tested. All four showed acquisition of erythromycin resistance, with an MIC value of 8 μg/ml. A representative transformant was named FP92, checked by DNA sequencing and used as a control in further experiments.

### The *msr*(D) Gene in *Streptococcus pyogenes*

The contribution of *msr*(D) to macrolide resistance was also studied in *S. pyogenes*, which is the original host of Φ1207.3 (Santagati et al., 2003). Two isogenic strains of *S. pyogenes* were constructed: (i) FR156, carrying Φ1207.3, and (ii) FR155, carrying Φ1207.3/Δ*msr*(D) (**Table 1**). Φ1207.3 transfer from *S. pneumoniae* donors to *S. pyogenes* recipients occurred at frequencies of 10**^-^**^7^ recombinants per donor. FR155 lacking *msr*(D) was susceptible to erythromycin (MIC of 0.12 μg/ml), whereas FR156 was resistant, with an MIC of 8 μg/ml (**Table 2**). These data show that in *S. pyogenes* the presence of the *mef*(A) gene alone determines a fourfold increase of the MIC for erythromycin, while when both *mef*(A) and *msr*(D) are present, the MIC is increased by 256-folds (**Table 2**).

The Φ1207.3/Δ*msr*(D) *S. pyogenes* FR155 was complemented *in trans* transferring a wild type Φ1207.3 element from FR122. Recombinants were selected for erythromycin resistance acquisition and in a representative recombinant, FR160, the presence of both the wild type and mutated form of Φ1207.3 was confirmed by DNA sequencing. FR160 showed an erythromycin MIC value of 8 μg/ml.

### Erythromycin Efflux

**FIGURE 3:**
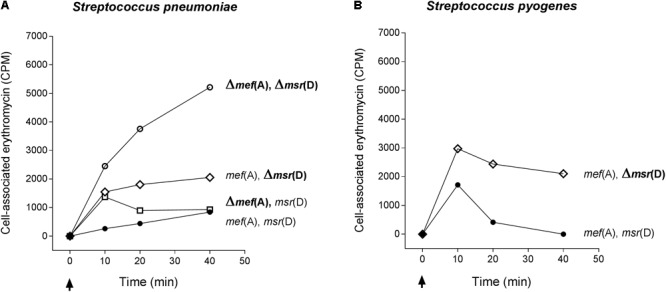
Efflux assay in *S. pneumoniae* **(A)** and *S. pyogenes* **(B)** isogenic mutant strains. The Δ*mef*(A)–Δ*msr*(D) *S. pneumoniae* mutant showed the maximum accumulation of radiolabeled erythromycin. Erythromycin efflux was not affected by the deletion of *mef*(A). *S. pneumoniae* and *S. pyogenes*Δ*msr*(D) mutants showed an intracellular accumulation of erythromycin peaking 10 min after erythromycin addition. Genotype is indicated on the right of each graph. Cell-associated erythromycin is reported as counts per minute (CPM). Arrows indicate the time point at which radiolabeled erythromycin was added. Full circles indicate the wild type strain carrying *mef*(A) and *msr*(D), open circles the *mef*(A) and *msr*(D) deletion mutant, open diamonds the *msr*(D) deletion mutant, and open squares the *mef*(A) deletion mutant. In all curves, the CPM baseline value at time 0 was subtracted from the CPM values of each time point.

The function of *mef*(A) and *msr*(D) was studied by testing incorporation of radiolabeled erythromycin in the deletion mutants (**Figure 3**). Radiolabeled erythromycin was retained/incorporated by strains lacking *msr*(D), while *msr*(D)-carrying strains showed erythromycin efflux. This was observed both in *S. pneumoniae* (**Figure 3A**) and in *S. pyogenes* (**Figure 3B**). Accumulation of radiolabeled erythromycin was maximum in the Δ*mef*(A)–Δ*msr*(D) *S. pneumoniae* mutant. The deletion of *mef*(A) did not affect erythromycin efflux: Δ*mef*(A) mutant showed an erythromycin accumulation comparable to that of the wild type strain, even if the kinetics of accumulation were different (**Figure 3A**). Both the *S. pneumoniae* and the *S. pyogenes*Δ*msr*(D) mutants showed an intracellular accumulation of erythromycin which peaked already 10 min after erythromycin addition (**Figures 3A,B**). These results were confirmed in a series of control efflux assays which included (i) testing deletion mutants derived a “*mef*(E)-positive strain” carrying *mef(*A)–*msr*(D) allelic variants and (ii) testing complemented strain FP92 (data not shown). Taken together, these results suggest that type M resistance to macrolides in streptococci is due to an efflux transport system of the ABC superfamily, in which *mef*(A) encodes the transmembrane channel, and *msr*(D) the two ATP-binding domains.

## Discussion

In this work, protein sequence analysis showed that Mef(A) contains 12 transmembrane segments and Msr(D) contains two ATP-binding domains (ABC domains). Analysis of bacterial genomes showed that *mef*(A) and *msr*(D) are found in tandem in 33 out of 37 cases. We constructed isogenic deletion mutants in *S. pneumoniae* and *S. pyogenes* to define the respective contribution of *mef*(A) and *msr*(D) to macrolide resistance. Physiological studies by MIC determinations and efflux assays, carried out in the original streptococcal hosts, showed that *msr*(D) is essential for erythromycin resistance and drug efflux, whereas *mef*(A) deletion produces a twofold reduction of MIC and does not affect efflux. Thus, it appears that *mef*(A) and *msr*(D) encode an ABC transporter involved in macrolide efflux with Mef(A) as the transmembrane channel, and Msr(D) as the cytoplasmatic ATP-binding protein. ABC transporters are multidomain membrane proteins minimally composed of two transmembrane domains and two ATP-binding domains. Transmembrane domains are responsible for binding and transport, while ATP-binding domains for coupling the energy of ATP hydrolysis to conformational changes in the transmembrane domains. These four domains may belong to a single protein or to different proteins.

In *E. coli*, the majority of transporters are secondary transporters and rely on proton-motive force as a source of energy^3^. Streptococci are fermentative organisms that use ATP as primary source of energy; accordingly, the majority of transporters are ATP-dependent. Because of these metabolic differences, it is likely that Mef(A) works as a major facilitator when expressed in *E. coli* (Clancy et al., 1996), while in the original streptococcal host, Mef(A) is coupled to the cognate ATP-binding protein Msr(D) to function as an efflux system.

In this work, we show that the *msr*(D) gene (i) is always in tandem with *mef*(A) (**Figure 1**), constituting a single transcriptional unit, and (ii) confers type M resistance to macrolides by an ATP-dependent efflux mechanism. The *msr*(D) gene product, Msr(D), is phylogenetically classified in the ABC-F family of ABC transporters together with other ATP-binding proteins, such as Vga and Lsa, which confer resistance also to other ribosome targeting antibiotics such as lincosamides, streptogramins, pleuromutilins, and ketolides. Proteins in this family share a general architecture with two NBDs and a long, mostly alpha helical, linker sequence connecting them. The spectrum of antibacterial resistances conferred is variable among members of this family, with some proteins giving resistance to multiple classes of antibiotics (Lenart et al., 2015; Sharkey et al., 2016).

Since deletion of *mef*(A) did not affect erythromycin efflux and produced only a twofold reduction on erythromycin MIC, it is possible that in the absence of Mef(A), Msr(D) uses an alternative transmembrane protein to pump the antibiotic out of the cell. A BlastP search in the genomes of *S. pneumoniae* TIGR4 and *S. pyogenes* M1 revealed that there are, respectively, three and one hypothetical transmembrane proteins homologous to Mef(A). Site-specific mutagenesis of the *mef*(A) homologous genes will be a first step for the identification of alternative Msr(D) cognate transmembrane domains which could complement the Mef(A) function.

## Author Contributions

FI and GPo designed the experiments. FI, FS, MS, J-DD, EL, GPa, and MC performed the experimental work. FI, FS, MO, GR, SS, and GPo analyzed and interpreted the data. FI, FS, and GPo wrote the paper.

## Conflict of Interest Statement

The authors declare that the research was conducted in the absence of any commercial or financial relationships that could be construed as a potential conflict of interest.

## Footnotes

1 http://www.tcdb.org/

2 https://blast.ncbi.nlm.nih.gov/Blast.cgi?PAGE_TYPE=BlastSearch&BLAST_SPEC=MicrobialGenomes

3 http://www.membranetransport.org/transportDB2
