# Supplementary material for: Type M Resistance to Macrolides Is Due to a Two-Gene Efflux Transport System of the ATP-Binding Cassette (ABC) Superfamily
Source: Front Microbiol. 2018 Jul 31;9:1670. doi: 10.3389/fmicb.2018.01670 (PMC6079230; doi:10.3389/fmicb.2018.01670)
Supplement: Supplementary file 1 [file Table_1.DOCX]

**TABLE S1.** Oligonucleotides used in this study

| Primer | Sequence, position on the submitted sequence (GenBank accession no.) |
| --- | --- |
| IF38 | ATG AAA ATT TGT TTG ATT TTT AAT GG, nucleotides  176 to 200 of the *ami* promoter (X17337) |
| IF184 | ATG AAC TTT AAT AAA ATT GAT TTA GA, nucleotides 1260 to 1285 ofthe pC194 plasmid (V01277) |
| IF39 | TTA TAA AAG CCA GTC ATT AGG CCT ATC T,  complementary to nucleotides 1883 to 1910 of the pC194 plasmid (V01277) |
| IF100 | GCT CTA GAA CTA GTG GAT C, nucleotides 1 to 16 of pR412 (AY334020) |
| IF101 | TTC CCT TCA AGA GCG ATA C, complementary to nucleotides 872 to 890 of  pR412 (AY334020) |
| IF105 | CTATGTCTTTAATATGAATGTTTCC, nucleotides 3112 to 3135 of Φ1207.3 (AY657002) |
| F35 | *CCA TTA AAA ATC AAA CAA ATT TTC AT*T GCC CTG CCC ATA TTG TAT AA the first 26 nucleotides are complementary to IF38, whereas the last 21 to nucleotides  3287 to 3307 of 3135 of Φ1207.3 (AY657002) |
| F183 | *AGA TAG GCC TAA TGA CTG GCT TTT ATA A*AT TTT AGG TAG TCG CTG GT, the first 28 nucleotides are complementary to IF39, the last 19 correspond to  nucleotides 6056 to 6074 3135 of Φ1207.3 (AY657002) |
| IF176 | ATG TAC TGG TTG CCT TTA ATA A, nucleotides 2155 to 2176 of 3135 of Φ1207.3 (AY657002) |
| IF182 | *TCT AAA TCA ATT TTA TTA AAG TTC AT*T TAT ATT ATC TCC TG, the first  26 nucleotides correspond to 1260 to 1285 of the pC194 plasmid (V01277), the last 15 nucleotides are complementary to 3240 to 3254 of 3135 of Φ1207.3 (AY657002) |
| IF185 | *AGA TAG GCC TAA TGA CTG GCT TTT ATA A*AC AAT ATT GGA GGG ATA TTT,the first 28 nucleotides are complementary to IF39, the last 20  nucleotides correspond to 4473 to 4492 of 3135 of Φ1207.3 (AY657002) |
| F20 | TAT TTT AAG CCT TGT TTC TTC ACC A, complementary to nucleotides 4897 to 4921 of 3135 of Φ1207.3 (AY657002) |
